# Alfalfa biotypes with putative enhanced cell wall digestibility and effects on performance of growing beef steers

**DOI:** 10.1093/tas/txac032

**Published:** 2022-03-09

**Authors:** Caleb W Karls, David K Combs, M Liou, Daniel M Schaefer

**Affiliations:** 1 Department of Animal and Dairy Sciences, University of Wisconsin-Madison, Madison, WI 53706, USA; 2 Department of Statistics, University of Wisconsin-Madison, Madison, WI 53706, USA

**Keywords:** apparent digestibility, cell wall, legume, lignin

## Abstract

Three alfalfa biotypes were chosen based on the presumption that they would be sources of alfalfa herbage that differed in lignin concentration and therefore cell wall digestibility. The hypothesis was that a lesser lignin concentration would result in greater alfalfa neutral detergent fiber (NDF) digestibility and greater beef steer growth performance. The three alfalfa biotypes were HarvXtra (Forage Genetics International), Hi-Gest 360 (Alforex Seeds), and a control alfalfa, LegenDairy XHD (Winfield Solutions LLC). High-moisture wrapped bales were prepared from second-harvest, d 30 crops. Digestibility of NDF was determined using in vitro incubations and a steer digestibility trial. Alfalfa baleage and trace mineral salt were fed to Angus steers (300 kg initial body weight, 4 pens/treatment) in an 83-day growing-phase trial. Alfalfa acid detergent lignin concentrations were 75.6, 71.8, and 63.0 g/kg dry matter (*P* = 0.34) for LegenDairy, Hi-Gest and HarvXtra, respectively. Based on in vitro total-tract NDF digestibility coefficients, HarvXtra tended (*P* ≥ 0.09) to have the highest NDF digestibility. Alfalfa biotype affected in vivo apparent total tract digestibility of NDF (*P <* 0.001) and there was a trend for an effect on acid detergent fiber digestibility (*P =* 0.051). Hi-Gest and HarvXtra had similar in vivo apparent NDF digestibilities, which were greater than for LegenDairy (*P <* 0.05). There was no alfalfa biotype effect on daily alfalfa dry matter intake (DMI; *P* = 0.51) or average daily gain (*P =* 0.25) by growing steers. The absence of an effect by the novel alfalfa biotypes on DMI by growing steers suggests that the compositional and digestibility differences of the novel alfalfa biotypes compared to LegenDairy were not sufficient to alleviate the limitation of physical fill (if evident) on DMI. If more disparity in cell wall composition and NDF digestibility were to exist between control and reduced-lignin biotypes, then perhaps an advantage in cattle growth performance for a reduced-lignin alfalfa biotype would be detectable.

## INTRODUCTION

Alfalfa (*Medicago sativa* L.) is one of the most widely grown crops in the world today (Barnes et al., 2003). Nearly 7.3 million ha of alfalfa are harvested in the United States alone (Fernandez et al., 2019). The value of alfalfa was estimated to be $10 billion USD in 2018, making it the third most valuable crop in the US behind corn and soybeans (Fernandez et al., 2019). Alfalfa is commonly harvested to produce alfalfa silage or hay and fed widely in lactating dairy cow diets and to a more limited extent in feedlot cattle diets in North America.

Alfalfa digestibility is limited due to the cell wall structural polyphenolic compound, lignin. Forage digestibility research has established a negative correlation between lignin concentration and fiber digestibility (Casler, 1987; Jung et al., 1997; Reddy et al. 2005). Oba and Allen (1999) discerned that an increase in neutral detergent fiber (NDF) digestibility in vitro or in situ was associated with increased dry matter intake (DMI) and milk production by lactating dairy cows.

Efficiency of alfalfa use has recently been re-considered in terms of plant digestibility and harvest efficiency. Scientists developed a genetically engineered alfalfa with a reduced lignin trait (Barros et al., 2019), which is the result of downregulating the gene encoding caffeoyl-CoA 3-*O*-methyltransferase (**CCOMT**). This novel alfalfa is thought to be more efficient because of increases in NDF digestibility (Grev et al., 2017; Getachew et al., 2018; Arnold et al., 2019). Reduced-lignin alfalfa is also considered more efficient because farmers could delay alfalfa harvest to increase forage yield or avoid inclement weather, while maintaining forage quality with only a 3-cut vs. 4-cut harvest regimen (Undersander et al., 2009). Another approach to improving alfalfa quality is to dilute the stem cell wall fraction (Albrecht et al., 1987) by genetic selection for an increased leaf-to-stem ratio.

The objective was to feed an alfalfa diet using alfalfa biotypes that presumably differed in NDF digestibility for the purpose of assessing biotype effects on cattle DMI and growth performance. It was hypothesized that the reduction in lignin concentration of alfalfa, harvested at an immature, constant sward age, and the associated increase in alfalfa NDF digestibility would result in increased DMI and increased average daily gain (ADG) of growing cattle.

## MATERIALS AND METHODS

All procedures involving cattle were approved by the College of Agricultural and Life Sciences Animal Care and Use Committee (protocol A005870) and followed the standards published in the ADSA-ASAS- PSA Guide for Care and Use of Agricultural Animals in Research and Teaching (2020).

### Alfalfa Biotypes and Agronomic Management

Three alfalfa biotypes were grown for digestibility evaluation and a steer growth performance trial. LegenDairy XHD was the high-quality control alfalfa (N313-16CVS, https://www.winfieldunited.com/products/winfield-united-seed/alfalfa/legendairy-xhd, Winfield Solutions LLC, St. Paul, MN), Hi-Gest 360 was a variety selected for high leaf-to-stem ratio (16-14913-CD, https://www.alforexseeds.com/alfalfa-products-2/alforex-seeds-alfalfa-varieties/hi-gest-360-alfalfa-seed/, Alforex Seeds, Woodland, CA), and HarvXtra RR 4.0 was a genetically-modified reduced-lignin alfalfa biotype (H103-16CVS, Winfield Solutions LLC, St. Paul, MN). HarvXtra RR 4.0 was a blend of early generation breeding lines produced for limited commercial launch in 2016 (Randy Welch, Croplan National Alfalfa Agronomist, personal communication). The fall dormancy ratings of LegenDairy and Hi-Gest were 3 and the blend of HarvXtra varieties was reported to be 4 (Randy Welch, Croplan National Alfalfa Agronomist, personal communication). Winter hardiness indices were 1.2, 1.5, and 2 for LegenDairy, Hi-Gest and HarvXtra, respectively.

In the spring of 2016, three separate fields were selected and planted at the University of Wisconsin-Madison Arlington Agricultural Research Station located near Arlington, WI (43°20ʹ N latitude, 89°22ʹ longitude, 320 m elevation). LegenDairy was planted on May 3, 2016 into a 6.8 ha field with a dominant soil type of Saybrook silt loam. Hi-Gest and HarvXtra were planted on May 5, 2016. The Hi-Gest field was 6.6 ha with a dominant soil type of Saybrook silt loam. The HarvXtra field was 6.7 ha with a dominant soil type of Plano silt loam, and adjacent to the Hi-Gest field. All alfalfas were planted with a John Deere 1590 drill (Deere & Company, Moline, IL) with row spacing of 19.05 cm. After accounting for pure seed, seed coating, germination and hard seed percentages, pure live seed of each biotype was planted at the rate of 8.0 kg/ha. The seed coating included a proprietary rhizobial inoculum and additional seed protectants, which were two fungicides, zinc, manganese and Ascend plant growth regulator (http://www.cdms.net/ldat/ldCFI002.pdf; Winfield Solutions LLC, St. Paul, MN). Field fertility was managed to exceed the minimum recommendations according to University of Wisconsin-Extension (Laboski and Peters, 2012). Fields were cut and chopped on July 10, 2016 (LegenDairy) and July 11, 2016 (Hi-Gest and HarvXtra), and August 22 and 23, 2016, respectively.

### Alfalfa Harvest and Storage

High-moisture baleages prepared from alfalfa swards harvested at a constant chronological age provided the alfalfa biomass. The second-cutting alfalfa crop was harvested in 2017 for use in the experiments. There were no herbicide or insecticide applications to the herbage that was harvested. Visual observations of the three alfalfa treatments prior to harvest indicated that fields had little to no nonalfalfa vegetation. The maturation state of the alfalfa plants was 10% bloom. All alfalfa treatments were disc-mowed, raked, and baled using the same machinery. Second cutting occurred on July 5, 2017, 30 d after first cutting, which is a typical interval for harvest of high quality alfalfa in the northern Midwest (Grev et al., 2017). Average daily air temperature and total precipitation during June 2017 were 20.1 °C and 15 cm, respectively; which were similar to the 10-yr average values of 18.9 °C and 14 cm, respectively. All fields were round-baled (122 × 152 cm, CIH RB455, Case Corporation, Racine, WI). Bales were wrapped contiguously with an in-line bale wrapper (H&S LW2, H&S Manufacturing Company Inc., Marshfield, WI) on July 6, 2017 using a continuous plastic sheet (Agriseal, Norflex Inc., Hudson, WI) of 1 mm thickness and applying seven wraps (Undersander, 2015). All treatments were wrapped in three parallel bale lines that were stored outside on the soil, and each bale line end was sealed. Bales were wrapped on the same day as baling and remained wrapped until needed for feeding. If a puncture to the wrap occurred, it was promptly sealed.

### Experimental Designs involving Cattle

The grow trial (**Trial G**) was designed to compare the three alfalfa biotypes fed to growing-phase beef steers using a randomized complete block design applied to 12 pens of cattle. There were four replicate blocks of pens for cattle fed each biotype, and pen was the experimental unit. Alfalfa baleage was fed as the sole source of digestible energy, consistent with the comment by Mertens and McCaslin (2008) that effects of reduced lignin alfalfas were greater when the alfalfas were fed in hay-only diets rather than total mixed rations.

Beef steers were used since the nutrient requirements of growing steers can be met by this sole source of protein, energy, macro-minerals and vitamins, and interpretation of results does not invoke proportionality of alfalfa in DMI. Seventy-two black Angus steers (approximately 250 kg body weight, BW) were purchased via auction from Bloomington Livestock Exchange, Bloomington, WI on December 8, 2017 for Trial G. All cattle were confirmed to not be persistently infected with bovine viral diarrhea virus, palpated to confirm castration, and injected with Dectomax (Zoetis Inc., Kalamazoo, MI) to remove gastrointestinal helminths, Bovi-Shield Gold 5 (Zoetis Inc., Kalamazoo, MI) for control of bovine respiratory disease, and Vision-7 (Intervet Schering-Plough, Somerville, NJ) to prevent clostridial diseases. Steers were housed 7 steers per pen (8.1 m^2^ per animal) for weight blocks 1 and 2, and 5 steers per pen (8.6 m^2^ per animal) for weight blocks 3 and 4. No anabolic implant was administered to steers. An automatic waterer was shared by each two adjacent pens. All pens were in a pole barn and bedded with sawdust to avoid bedding consumption. Prior to feeding alfalfa baleage, steers were fed dry grass hay ad libitum. Steers were transitioned to a nonexperimental alfalfa baleage 5 d prior to d 0 of trial G to alleviate pre-trial gut fill as a source of variation in initial BW. On d 0, steers were weighed individually prior to feeding and stratified by d 0 BW. Steers were randomly allocated by stratum within block to five steers per pen for six pens and seven steers per pen for six pens. Alfalfa treatments were randomly allocated to pens within block. Two consecutive prefeeding morning weights were taken for each steer for initial (d 0 and 1) and final full BW. The duration of Trial G was 83 d because the quantity of baleage harvested and the feed intake of the steers did not allow for the intended trial duration of 90 d. HarvXtra was the first biotype bale supply to be exhausted.

Concurrent with Trial G, 6 black Angus steers (310 ± 5 kg, mean ± SD) were used for a digestibility trial (Trial D). Using a design with two replicated 3 × 3 Latin squares (Latin rectangle), each steer was fed one of the three alfalfa biotypes in each period for a series of three periods for a balanced crossover study. Each period lasted 14 d and during the final 3 d of each period, all feces were quantitatively collected using a harness and fecal collection bag (Nelson Weaver Enterprises, Dalton, WI).

### Feed Management

Alfalfa bales were processed through a bale chopper (Teagle 8080, Teagle Machinery Ltd, Three Burrows, Truro, UK) with knives set to minimum penetration, prior to feeding to cattle. Disentanglement and not particle size reduction of baleage was the strategy. A bale from each biotype was chopped as needed, and resulted in approximately 2 d of forage supply. Since Trial G occurred between February 7, 2017 and May 1, 2017, the prevailing ambient temperature minimized baleage spoilage after chopping. Each pen was fed alfalfa baleage ad libitum once per day in a fenceline bunk at 0800 h. Daily allotments of chopped baleage were adjusted to have no more than 2 kg per pen of alfalfa orts after a 24 h period. Bunks were filled manually by using a pitch fork to move alfalfa from a chopped baleage biotype pile into a feed cart, which was weighed on a platform scale, and then again baleage was pitch-forked into the respective pen bunk. Each bunk had one 22.7 kg trace mineral salt block (Champion’s Choice, Cargill Inc., Minneapolis, MN). Guaranteed analysis of the salt block was as follows (mg/kg): 940,000–990,000, NaCl; 3,500, Zn; 2,000, Fe; 2,000, Mn; 300, Cu; 70, I; and 50, Co. Each trace mineral block was weighed at the beginning and end of trial G to calculate total consumed trace mineral salt per pen.

### Sampling

The total number of bales fed for each treatment was as follows: LegenDairy, 58; Hi-Gest, 58; and HarvXtra, 62. Three core samples (approximately 100 g total) per bale were taken for subsequent nutritional analysis before each bale was chopped. Core samples were obtained using a hay probe (2 cm diameter and 43 cm length) mounted to an electric drill. Samples were immediately bagged and frozen (−14 °C) for future analysis. Approximately 50 g per fed bale were composited over a 4-wk interval. A total of three composite samples per alfalfa biotype were generated. Each composite sample was mixed, and a subsample was sent to a commercial laboratory for wet chemistry analysis (Rock River Laboratory, Watertown, WI, USA).

### Digestibility Trial

During Trial D, steers were housed in individual pens (12.2 m^2^ per animal). An automatic waterer was shared by each two adjacent pens. All pens were in a pole barn and bedded with sawdust to avoid bedding consumption. Prior to feeding alfalfa baleage, steers were fed a corn silage basal diet. Steers were transitioned to a nonexperimental alfalfa baleage-only diet 5 d prior to d 0. Each steer had ad libitum access to a trace mineral salt block (Champion’s Choice, Cargill Inc., Minneapolis, MN) during the course of Trial D.

The feces of each steer were collected, weighed, mixed, sampled (10% of the total) and refrigerated (5 °C) every 12 h for 3 d. At the end of each sampling period, these samples were mixed and a sample of 2400 g was retained per steer and frozen (−14 °C) for future analyses.

The alfalfa baleage fed to the trial D steers was from the same 2017 harvest and bales as were fed to the Trial G steers. Trial D occurred between March 23, 2017 and May 3, 2017. A 0.5 kg grab sample of chopped alfalfa was retained during feeding for subsequent analysis per steer per period for d 11, 12, and 13. Feed refusal was removed from individual bunks and weighed on d 14 of each period. Grab samples of refused feed were taken for each individual steer by random grabs until a total of 0.5 kg was reached. Feed and refusal samples were bagged and frozen immediately for future analysis. Before analysis, frozen feed samples were composited while in the frozen state by steer and period. Composite subsamples were sent to a commercial laboratory for wet chemistry analysis (Rock River Laboratory, Watertown, WI, USA).

### Analytical Methods of Rock River Laboratory

All samples were dried and ground to pass 1 mm screen (Udy Cyclone, Udy Corp., Fort Collins, CO, USA). NDF was determined using method 6 (Ankom Technologies, 2017a), which includes addition of sulfite and heat-stable amylase but not an ashing step. Acid detergent fiber (ADF) was determined using method 5 (Ankom Technologies, 2017b). Acid detergent lignin (ADL) was determined in ADF residue samples after sulfuric acid digestion (method 9, Ankom Technologies, 2020). The methods for CP (AOAC 990.03), ether extract (AOCS Am5-04, Rapid Determination of Oil/Fat Utilizing High-Temperature Solvent Extraction; AOCS, 2017), starch (Hall, 2009), ash (AOAC 942.05) and minerals (Modified AOAC 968.08) are reported at https://docs.google.com/spreadsheets/d/1Mmf7v-wT5GDbho1kbO2PzEPUxut0IzgtRO6XU-Ed-JE/edit?u sp=sharing and by AOAC (2003). Neutral detergent-insoluble crude protein and acid detergent-insoluble crude protein (ADICP) were determined by doing a Dumas combustion (AOAC 990.03; AOAC, 2003) of NDF and ADF residues, respectively, followed by nitrogen determination and then multiplying nitrogen times 6.25. Available CP is CP less ADICP. Nonfiber carbohydrate was calculated using the method of Lanzas et al. (2007). The relative forage value index was calculated according to the logic described by Undersander et al. (2010). Net energy values for maintenance and gain were calculated using equations from the dairy NRC (2001).

### In vitro NDF Digestibility

In vitro analyses were conducted on subsamples of the three 4-wk composite samples for each alfalfa biotype from Trial G. Each composite sample was an experimental unit. The methods of Lopes et al. (2015) were used for the in vitro analysis of alfalfa samples and calculation of total-tract NDF digestibility (**TTNDFD**). Briefly, in vitro incubation of triplicate dried (60 °C), ground (1 mm) alfalfa samples (0.5 g) in F57 filter bags (Ankom Technology, Macedon, NY) followed the method of Goeser and Combs (2009) using rumen fluid collected and pooled from two rumen-cannulated, lactating Holstein cows that were fed a 95% forage diet. After collection, the rumen fluid inoculum was primed with a mixture of 40% cellulose, 20% urea, 20% corn starch, and 20% cellobiose (Goeser et al., 2009) and allowed to incubate until achieving 0.1 mL of gas production/mL of rumen fluid. In vitro incubations were conducted with triplicate bags of each sample, duplicate empty filter bags to generate the bag particle influx correction factor, and a sample of each of two standard ground, alfalfa hays to assess inter-assay variability, though these results were not used as a correction factor. Samples were incubated at 39 °C with agitation and terminated at 0, 24, 30, 48, and 240 h. Subsequent determination of NDF followed the method described by Lopes et al. (2015) which followed method 6 (Ankom Technologies, 2017a). Results from 0 h samples represented total NDF, and 240 h sample results represented indigestible NDF (**iNDF**). Results at each incubation time point were expressed as proportion of potentially digestible NDF (**pdNDF**) that disappeared. Digestion rates (*k*_d_) were the negative value of slope for log_e_ (1 – NDF digested/pdNDF) versus incubation time using the SLOPE function of Excel (Microsoft Office Professional Plus 2016). The TTNDFD coefficients were calculated as follows:


$$\begin{array}{l} \begin{array}{ll} & pdNDF=1NDF_{240\;h},g/kg\;NDF\\ & TTNDFD,\;g/kg\;NDF=pdNDF\times \left(k_{d}/\left(k_{d}+k_{p}\right)\right)/0.9 \end{array} \end{array}$$

Particle passage rate (*k*_p_) was predicted as follows from the regression model of Krizsan et al. (2010): Particle passage rate (*k*_p_, h^−1^) = (*F* + 1.54 + 0.0866 × NDF intake in g/kg BW)/100, where *F* = 0.24, due to alfalfa as sole forage component.

### Statistical Methods

Animal performance data from Trial G were analyzed as a randomized complete block design using the MIXED procedure of SAS (SAS 9.4, SAS Institute Inc., Cary, NC), The model was as follows:


$$\begin{array}{l} Y_{jk}=\mu +T_{j}+b_{k}+e_{jk}, \end{array}$$

where *Y*_*jk*_ = response variable; *µ* = grand mean; *T*_*j*_ = the fixed effect of alfalfa biotype; *b*_*k*_ = the random effect of initial BW block; and *e*_*jk*_ = experimental error. Pen was the experimental unit. Trial D data were analyzed as a three period by six steer, Latin rectangle, crossover study with three unique treatment sequences using a mixed model in the MIXED procedure of SAS. In this design, we opted for more power in detecting existence of a carryover effect versus estimating all carryover effects in a balanced manner. The model without carryover effects is symbolically described as follows:


$$\begin{array}{l} Y_{jkl}=\mu +P_{j}+T_{k}+s_{l}+e_{jkl}, \end{array}$$

where *Y*_*jkl*_ = response variable; *µ* = grand mean; *P*_*j*_ = the fixed period effect; *T*_*k*_ = the fixed effect of alfalfa biotype; *s*_*l*_ = the random effect of steer subject, and *e*_*jkl*_ = experimental error. Animal by period was the experimental unit. The link function between response and systematic component was chosen to be the identity function (gaussian) by visual inspection of the residuals. A bias-corrected Kenward-Roger precision matrix estimator (KR2) was used for degree of freedom estimation in all mixed models (Kenward and Roger, 2009). The covariance matrix within steer was modeled as “unstructured” (UN) for all response variables, which was selected by likelihood-based Bayesian information criteria from among several other common covariance structures including autoregressive (1), ante-dependent, compound symmetry (CS), and Toeplitz. Covariance structure modeling was also validated visually and by nested model likelihood ratio tests (CS vs. UN *P* = 0.000822). The UN covariance structure was also selected by Bayesian information criteria when responses were studied with an additive effect for each type of response nested within the steer. Statistical carryover effects of the design were tested for all responses by nested model likelihood ratio tests. Overall, there was little evidence for a carryover effect from treatments. A “pdmix800” macro (Saxton, 1998) was used to generate letter plots for treatment groups at the α = 0.05 level.

Nutritional characteristics and in vitro data were analyzed as a randomized complete block design using the MIXED procedure of SAS and the following model:


$$\begin{array}{l} Y_{jk}=\mu \;+T_{j}+m_{k}+e_{jk}, \end{array}$$

where *Y*_*jk*_ = response variable; *µ* = grand mean; *T*_*j*_ = the fixed effect of alfalfa biotype; *m*_*k*_ = the random effect of composite sample month; and *e*_*jk*_ = experimental error. A 4-wk composite sample was the experimental unit. When the *F*-statistic was significant (*P* ≤ 0.05), the LSMEANS statement with the pdmix800 macro (Saxton, 1998) was used to show treatment groupings at the α = 0.05 level. Tendencies were reported for *P*-values (0.10 ≥ *P* > 0.05).

## RESULTS AND DISCUSSION

Alfalfa biotypes were chosen for this project on the basis of their common use in commercial production (LegenDairy), anticipated dilution of stem lignin in harvested herbage (Hi-Gest), and genetic down-regulation of lignin biosynthesis (HarvXtra). In preliminary research, we harvested these biotypes at d 40 of chronological age as second-cutting alfalfa in 2016. We learned that there was no difference in ADL concentration due to alfalfa biotype at that age. Consequently, we chose d 30 of chronological age for this project, and second cutting to avoid harvest of any fall growth, to lessen the risk of grass growth in the sward, and to assure uniformity of plant re-growth initiation. Across a wide variety of alfalfa cultivars, ADL concentration is less at a 30-d, rather than 40-d, cutting interval (Sulc et al., 2021).

The context for this project design was the harvest of early maturity, excellent quality alfalfa with presumed differences in ADL content. Compositional results for the alfalfa biotypes are shown in Table 1. There was no visual evidence of mold in the alfalfa biotypes and the cattle readily ate their respective biotype. Hi-Gest and HarvXtra baleages were more dry (*P <* 0.05) with greater crude protein (CP) and available CP concentrations (*P <* 0.05) than the LegenDairy treatment. The differences in dry matter (DM) concentration did not result (*P =* 0.24) in an effect on acid detergent insoluble protein, for which concentrations were very low. The organic matter content did not differ (*P* = 0.39) among biotypes. Arnold et al. (2019) associated mean stage count, i.e., plant maturity, with ash concentration. Mean stage count was not determined here, but using the logic of Arnold et al. (2019) there appear to be no differences in plant maturity among the three biotypes since ash content was not affected by biotype. Alfalfa biotype did not have an effect (*P* > 0.34) on NDF, ADF, ADL and ether extract concentrations. Neutral detergent insoluble CP concentration was greater in Hi-Gest and HarvXtra than LegenDairy (*P* ≤ 0.05). While HarvXtra had the lowest numerical ADL concentration, its failure to reach statistical significance is not surprising considering the relatively large analytical error associated with the ADL assay. Hi-Gest and HarvXtra treatments had greater starch concentrations than LegenDairy (*P <* 0.05), but there were no differences (*P* = 0.69) in nonfiber carbohydrate concentration among biotypes. While there were differences among biotypes in mineral concentrations (Table 1), all concentrations were adequate relative to nutrient requirements for growth-phase beef steers (NASEM, 2016). Relative forage value indices (Undersander et al., 2010), total digestible nutrients, and energy values expressed in terms of NE_m_ and NE_g_ did not differ (*P >* 0.34) among alfalfa biotypes. The low proportion of ruminal undegradable protein (RUP, 0.15, Appendix Supplementary Table A2) is consistent with previous discussion of high-quality alfalfa (Broderick, 1995).

**Table 1.** Nutrient composition of three alfalfa biotypes harvested on d 30 of second-cutting maturity in 2017. Three composite samples composed of baleage core samples for each biotype were analyzed. Each composite sample included approximately 20 bales, each of which had been core-sampled three times.

Item	LegenDairy	Hi-Gest	HarvXtra	SEM	*P*-value
Dry matter (DM), g/kg	459^c^	549^a^	516^b^	7.2	<0.01
Component, g/kg DM					
Organic matter	886	894	891	4.8	0.39
Crude protein	185^b^	202^a^	196^a^	2.1	<0.01
ADICP^1^	7.1	7.3	6.8	0.2	0.24
Available CP^2^	178^b^	194^a^	189^a^	2.0	<0.01
NDF^3^	446	455	445	13.1	0.79
NDICP^4^	8.6^b^	12.1^a^	12.5^a^	0.56	<0.01
ADF^5^	383	386	376	10.1	0.58
ADL^6^	75.6	71.8	63.0	7.6	0.34
Ether extract	20.2	18.1	20.9	2.3	0.50
Starch	22.1^c^	27.3^b^	33.9^a^	0.8	<0.01
NFC^7^	244	231	238	13.9	0.69
Calcium	9.4^a^	8.6^b^	9.6^a^	0.1	<0.01
Phosphorus	3.3^a^	2.9^b^	3.1^a^	0.1	0.02
Magnesium	2.0^b^	2.2^a^	2.3^a^	0.1	0.03
Potassium	28.9^a^	26.9^b^	26^c^	0.3	<0.01
Sulfur	1.7^c^	2.4^a^	2.2^b^	0.1	<0.01
RFV^8^	123	121	124	3.7	0.70
TDN^9^	556	563	579	13.7	0.34
Net energy, Mcal/kg DM					
NE_m_^10^	1.17	1.21	1.28	0.07	0.34
NE_g_^11^	0.616	0.638	0.704	0.04	0.34

Means in a row without common superscripts differ at *P* ≤ 0.05.

Acid detergent-insoluble crude protein.

Available Crude Protein = Crude Protein – ADICP.

Neutral detergent fiber with addition of sulfite and heat-stable amylase.

Neutral detergent-insoluble crude protein.

Acid detergent fiber.

Acid detergent lignin; Lignin plus ash after sulfuric acid digestion.

Non-fiber carbohydrate; EE is ether extract; NFC = 100 – [(NDF – NDICP) + CP + EE + ash].

Relative forage value; 100 = full-bloom alfalfa.

Total digestible nutrients; td is truly digestible; tdNFC = 0.98 NFC; tdCP = CP * exp[-1.2 * (ADICP/CP)]; NDFn = NDF – NDICP, L is acid detergent lignin, tdNDF = 0.75 * (NDFn – L) * [1 – (L/NDFn)^0.667^; TDN = tdNFC + tdCP + [(EE – 1) * 2.25] + tdNDF – 7; equations 2-4a, 2-4b, 2-4e and 2–5, Dairy NRC (2001)

Net energy for maintenance; DE (Mcal/kg) = 4.409 * TDN, equation 2-1; ME (Mcal/kg) = 1.01 * DE – 0.45, equation 2-2; NE_m_ = 1.37 ME – 0.138 ME^2^ + 0.0105 ME^3^ – 1.12; equation 2–13, Dairy NRC (2001); MJ/kg = 4.184 MJ/Mcal * Mcal/kg.

Net energy for gain; NE_g_ = 1.42 ME – 0.174 ME^2^ + 0.0122 ME^3^ – 1.65; equation 2–14, Dairy NRC (2001).

The CP, NDF, and ADF concentrations and relative forage value index indicate that the harvested alfalfas did not qualify as excellent quality alfalfa such as is fed to high-producing lactating dairy cows. Winning entries in the World Dairy Expo Forage Super Bowl have 26–28% CP (Schwab and Broderick, 2017) and Premium grade alfalfa has a relative forage quality index greater than 170 (https://www.idahohay.com/uploads/1/7/5/2/17522397/hay_standards.pdf). Relative forage value and relative forage quality indices, although calculated somewhat differently, are both normalized so that an index value of 100 corresponds to full-bloom alfalfa. The modest CP concentrations are unlikely to be due to leaf loss since methods of harvest, preservation and feeding employed here did not result in disproportionate leaf loss. Harvest at mid-bud stage (Undersander, 2015) would have been beneficial though quantity of alfalfa herbage would have been less.

Although we observed no statistically significant effect of alfalfa biotype on ADL concentration (Table 1), the numerical difference in ADL concentration (HarvXtra vs LegenDairy, −12.6 g/kg DM) was greater than previously reported for HarvXtra-008 (Arnold et al., 2019) and referenced controls (−6.5 g/kg DM). Compared to LegenDairy, ADL concentrations for Hi-Gest and HarvXtra were 95% and 83%, respectively. Donnelly et al. (2018) sampled alfalfa fields on the Arlington Agricultural Research Station at five time points during 2017 first crop growth and found a slightly lower lignin concentration (−10.4 g/kg DM) in stems of HarvXtra vs. Hybriforce 3400. Donnelly et al. (2018) likewise found the stem ADL concentration of Hi-Gest 360 to be 95% of HybriForce. Although Hi-Gest has been selected for increased leafiness, the similarity of ADL concentrations between LegenDairy and Hi-Gest aligns with the results of Albrecht et al. (1987) who reported no difference between alfalfa stem and leaf lignin concentrations. Cherney et al. (2020) compared reduced-lignin alfalfa biotype Hx14376 to WL 355.RR across flower and bud stages at three U.S. locations and found the reduced-lignin biotype to have 14.1% less lignin, and Grev et al. (2020) reported that the lignin reduction in reduced-lignin alfalfa biotype 54HVX41 was due to decreased lignin in the stem and not leaf fraction. In general, HarvXtra has about 15% less lignin than varieties that have not received the CCOMT modification. Our ADL results are consistent with prior reports for biotypes denoted as reduced-lignin or having increased leafiness.

Three factors govern the digestible energy contribution of plant cell walls in the ruminant diet. They are quantity of potentially digestible NDF consumed, rate of NDF digestion, and rate of undigested NDF passage out of the rumen (Allen and Mertens, 1988). These factors can be determined experimentally or estimated from the literature, and integrated via the calculation of TTNDFD (Combs, 2013; Lopes et al., 2015).

Total-tract NDF digestibility (Combs, 2013; Lopes et al., 2015) was calculated for the alfalfa biotypes (Table 2). Concentration of NDF was not affected by biotype (*P* = 0.35), consistent with results shown in Table 1. In vitro-digested NDF at the sampling times was also not different among biotypes, though there was a tendency (0.09 ≥ *P* ≥ 0.06) for less NDF to be digested at each incubation time for the LegenDairy biotype. Table 2 shows that the concentration of iNDF was greater (*P <* 0.05) for LegenDairy than for Hi-Gest (−16%) and HarvXtra (−22%). The pdNDF fraction was less for LegenDairy than for Hi-Gest and HarvXtra; however, the digestion rate (*k*_d_) of pdNDF was faster (*P <* 0.05) for LegenDairy and similar to that of HarvXtra. The aggregation of these results culminated in TTNDFD coefficients that were not affected (*P* = 0.099) by biotype (Table 2) though there was a tendency for HarvXtra to have the largest TTNDFD coefficient.

**Table 2.** Determination of in vitro total-tract NDF digestibility (TTNDFD) coefficients for three alfalfa biotypes harvested on d 30 of second-cutting maturity. The biotype composite samples utilized here were identical to those analyzed for Table 1 results.

	LegenDairy	Hi-Gest	HarvXtra	SEM	*P*-value
NDF, g/kg DM	437	439	446	5.7	0.35
NDF digested, g/kg NDF					
24 h	176	207	195	9.1	0.06
30 h	219	249	264	15.3	0.09
48 h	352	373	415	21.4	0.09
iNDF^1^, g/kg NDF	568^a^	489^b^	467^b^	13.4	<0.01
*k*_d_ of pdNDF^2^, h^-1^	0.050^a^	0.034^b^	0.044^a^	0.0034	0.02
TTNDFD coefficient^3^	0.315	0.316	0.370	0.021	0.10

Means in a row without common superscripts differ at *P*≤ 0.05.

indigestible neutral detergent fiber, based on NDF remaining after 240 h of in vitro incubation.

Rate of digestion of potentially digestible NDF (pdNDF) based on in vitro incubations of 24, 30 and 48 h. *k*_d_ is negative value of slope for log_e_ (1 – NDF digested/pdNDF) vs incubation time.

TTNDFD = ((1000-iNDF) * (*k*_d_/(*k*_d_ + *k*_p_)))/0.9 in which *k*_p_ was calculated according to method of Krizsan et al. (2010) using NDF intake of Trial G steers, i.e., 9.51, 10.19, and 10.04 g NDF/kg BW for LegenDairy, Hi-Gest, and HarvXtra, respectively. Particle passage rate (*k*_p_, h^−1^) = (*F* + 1.54 + 0.0866 × NDF intake in g/kg BW)/100, where *F* = 0.24.

Trial D was utilized to characterize the in vivo apparent digestibility of the alfalfa cell walls for the biotypes fed during Trial G. Dry matter intake was greater for Hi-Gest than LegenDairy (*P <* 0.05) and HarvXtra was intermediate (Table 3). Apparent DM digestibility tended (*P* = 0.07) to be greater for HarvXtra than LegenDairy. Digestibility coefficients for NDF in Hi-Gest and HarvXtra were greater (*P <* 0.05) than for LegenDairy. There was a tendency (*P* = 0.051) for Hi-Gest and HarvXtra to also have greater ADF digestibility coefficients than LegenDairy. These results suggest that the NDF fraction of Hi-Gest and HarvXtra were similarly digestible and more digestible than the NDF of LegenDairy.

**Table 3.** Dry matter intake (DMI) and apparent total tract digestibility coefficients of dry matter (DM) and cell wall components in three alfalfa biotypes at d 30 of second-cutting maturity^1^ (Trial D).

Item	LegenDairy	Hi-Gest	HarvXtra	SE	*P*-value^2^
DMI, kg/d	6.49^b^	6.87^a^	6.66^ab^	0.22	0.02
DM	0.618	0.638	0.656	0.008	0.07
NDF	0.524^b^	0.580^a^	0.589^a^	0.009	<0.001
ADF	0.511	0.588	0.597	0.020	0.051

Means in a row without common superscripts differ at *P* ≤ 0.05.

Six Angus steers (310 ± 5 kg) were used in a digestibility experiment that was a 3 × 6 Latin rectangle. The Latin rectangle consisted of three periods of 14 d with fecal collection on d 12–14.

*P* values are overall Type 3 treatment effect *F*-tests.

Comparison of the TTNDFD estimates (Table 2) with the apparent NDF digestibility coefficients (Table 3) indicates lack of numeric agreement, for which there may be two possible explanations. One explanation may be the effect of a mixed forage-concentrate diet vs. a solely forage diet, as used in Trial D. The TTNDFD model of Combs (2013) is based on mixed forage-concentrate diets fed to dairy cows (Lopes et al., 2015). However, Lund et al. (2007) summarized that forage-only diets were associated with increased *k*_d_ and decreased *k*_p_ as compared to supplemented forage diets, and that this effect was especially evident for alfalfa hay or early cut grass silage vs. corn silage or late-cut grass silage. Second, Lund et al. (2007) mathematically discerned that particles of pdNDF are selectively retained in the rumen. For alfalfa hay, the *k*_p_ of iNDF was 0.017 h^−1^ and for digestible NDF, *k*_p_ was 0.0069 h^−1^ (Lund et al., 2007). Their alfalfa hay had similar composition and *k*_d_ (0.045 h^−1^) as the alfalfa biotypes fed here, though with greater proportion of lignin and lower proportion of iNDF. The TTNDFD model (Combs, 2013) was introduced with an assumed *k*_p_ for a lactating dairy cow (0.0267 h^−1^) fed a forage-concentrate diet, and 90% of pdNDF digestion was assumed to occur in the rumen. The TTNDFD values in Table 2 include the adjustment in *k*_p_ based on alfalfa NDF intake according to the equation of Krizsan et al. (2010; see Table 2, footnote 3). The resulting *k*_p_ values were 0.0260, 0.0266, and 0.0265 h^−1^ for LegenDairy, Hi-Gest, and HarvXtra, respectively, and yet the TTNDFD coefficients are 0.2 units less than apparent NDF digestibility coefficients (Table 3). However, the Krizsan et al. (2010) equation was the result of a meta-analysis and not developed with the approach of Lund et al. (2007). Therefore, the results of Lund et al. (2007) for a solely alfalfa hay diet appear to be the more relevant information. When the alfalfa *k*_p_ = 0.0069 h^−1^ (Lund et al., 2007) was introduced into the TTNDFD digestibility equation (Combs, 2013) and the ruminal digestion of pdNDF was assumed to be 98% (hay-only diet, Huhtanen et al., 2007), the mean TTNDFD values were 0.387, 0.433, and 0.470 g dNDF/g NDF for LegenDairy, Hi-Gest, and HarvXtra, respectively, and still lacked agreement with the NDF digestibility coefficients in Table 3. The TTNDFD calculation underestimated apparent NDF digestibility by growing steers fed alfalfa as the sole NDF source.

Inferences regarding the nutritional merit of reduced lignin concentrations in alfalfa thus far have been based mainly on compositional analyses using near-infrared reflectance spectroscopy (NIRS), an in vitro experiment, and three in vivo experiments. Grev et al. (2017) and Arnold et al. (2019) showed that two different HarvXtra varieties had greater NIRS-based NDF digestibilities when compared to reference alfalfa varieties. Getachew et al. (2018) reported a similar NIRS-based NDF digestibility advantage (7%), and furthermore used in vitro rumen gas production results to estimate that reduced lignin alfalfa biotypes had 5% more gas production, an indirect indicator of DM digestibility, and thus a 4% greater calculated metabolizable energy value. The results of Getachew et al. (2018) were derived from alfalfa lines with downregulated lignin synthesis that were harvested in West Salem, WI, in 2014, a site with climatic similarity to the conditions reported here. Alfalfa hay that was transformed by downregulating CCOMT was fed to lambs as the sole diet ingredient (Mertens and McCaslin, 2008). This alfalfa biotype had less lignin compared to its nontransformed control (52 vs. 59 g/kg DM, respectively; 12% reduction) and a greater NDF digestibility coefficient (0.501 vs. 0.464, respectively; 8% improvement). The effect of the CCOMT transformation on NDF digestibility was less when the alfalfa hay was fed as an ingredient in a total mixed ration (Mertens and McCaslin, 2008). Weakley et al. (2008) fed this CCOMT alfalfa biotype and its nontransformed control to lactating dairy cows and found no effect on DMI yet a greater NDF digestibility coefficient (0.486 vs. 0.445, respectively; 9% improvement). Getachew et al. (2011) evaluated the CCOMT genetic modification in field-grown alfalfa and reported similar magnitude of effects compared to its nontransformed control, i.e., 13% reduction in lignin, 6% more in vitro rumen gas production, and 4% greater in vitro DM digestibility. Peterson et al. (2018) fed HarvXtra or conventional alfalfa hays of premium quality to growing-phase Angus heifers. Lignin in their HarvXtra was reduced only by 4.2 g/kg (5.5% reduction) and there was no HarvXtra effect on alfalfa DMI or ADG. In general, the CCOMT transformation, such as was used in development of HarvXtra, appears to have a larger percentage unit effect on lignin reduction than NDF digestibility improvement, acknowledging the variability associated with measurement of both fractions. In general, the in vivo results highlight the insecurity of using compositional analyses and in vitro results to extrapolate to an in vivo performance benefit.

The results for growth performance of steers fed the three alfalfa treatments are shown in Table 4. There was no effect of biotype on ADG, alfalfa DMI or BW gain efficiency (*P* ≥ 0.25; Table 4). Trace mineralized salt intake was likewise unaffected (*P* = 0.29). Trial G steers were capable of faster BW gain, since similar steers fed a protein adequate, 72% corn silage-25% distillers grain diet had daily BW gain of 1.27 kg/d (Karls, 2020). Based on the average BW during the trial, alfalfa DM consumption was 21.3, 22.4, and 22.6 g/kg BW for LegenDairy, Hi-Gest and HarvXtra, respectively, and alfalfa NDF consumption was not different (*P* = 0.44) among the biotypes, corresponding to 9.5, 10.2 and 10.0 g NDF/kg BW, respectively. In the absence of a biotype effect on ADG, there was no evidence to suggest a difference among biotypes in net energy availability. Nevertheless, the steers were capable of growing more rapidly if a higher energy diet would have been provided. Clearly, net energy supply limited the ADG of these steers.

**Table 4.** Body weights (BW) and growth performance of beef steers fed three alfalfa biotypes harvested at d 30 of second-cutting maturity^1^ (Trial G)

	LegenDairy	Hi-Gest	HarvXtra	SEM	*P*-value^2^
Initial BW, kg/steer	300	300	300	0.87	0.64
Final BW, kg/steer	378	383	389	6.51	0.28
ADG^3^, kg/(steer × d)	0.94	1.00	1.07	0.07	0.25
Alfalfa DMI^4^, kg/(steer × d)	7.23	7.65	7.77	0.47	0.51
Gain efficiency^5^	0.129	0.132	0.139	0.014	0.78
Salt intake, g/(steer × d)	35.8	19.3	28.4	6.68	0.29
NDF intake, kg/(steer × d)	3.22	3.48	3.46	0.21	0.44

Black Angus steers were fed a diet consisting of solely alfalfa baleage plus trace mineral salt for 83 d.

*P* values are overall Type 3 treatment effect F-tests in a randomized complete block design with 4 blocks.

Average daily gain.

Alfalfa DMI was based on as-fed baleage weight disappearance from feed bunks times dry matter decimal determined (105 °C) for the three composite baleage samples per biotype.

Gain efficiency was calculated as ADG/alfalfa DMI.

With regard to the hypothesis of this project, we failed to detect an effect of alfalfa biotype on whole plant lignin concentration, therefore the implications of reduced lignin concentration on in vivo NDF digestibility and digestible energy availability to growing cattle cannot be discerned from these experiments. Nevertheless, the results presented here can provide helpful guidance to future examination of in vivo effects of reduced-lignin alfalfa when fed to cattle.

Trial G was designed to use growing-phase beef steers for which macro-nutrient requirements, except digestible energy, were met by feeding solely alfalfa baleage. In this context, biotype effects on daily gain or gain efficiency would be interpreted to be the result of differences in availability of digestible energy. To evaluate the validity of this premise, the metabolizable protein (MP) required by these steers and supplied by the alfalfa biotypes was calculated (Appendix Supplementary Table A2). The MP requirements were 568, 586, and 607 g MP/d, respectively, for LegenDairy, Hi-Gest and HarvXtra. The ruminal daily requirement for RDP is considered (NASEM, 2016; p 94) to be equal to rumen microbial protein daily synthesis. Since alfalfa in general and these biotypes specifically had RDP coefficients of approximately 0.85, the RDP supplied (1140–1300 g/d) far exceeded microbial CP synthesis (392–434 g/d, Appendix Supplmentary Table A2). The calculated MP supply was less than the total MP requirement by 152, 111, and 140 g/d for LegenDairy, Hi-Gest and HarvXtra, respectively. Thus, MP supply may have limited ADG for all biotypes.

Future evaluations of alfalfa cell wall digestibility in growing cattle should consider inclusion of dietary RUP supplementation, as has been noted for lactating dairy cow diets (Broderick, 1995). Dhiman et al. (1993) fed alfalfa silage having 17–22% CP to lactating dairy cows and alfalfa silage was 98% of their diet. When casein or soy protein was infused into the abomasum, milk yield and milk protein yield increased, leading them to conclude that protein, not energy, was first-limiting for lactating cows fed all forage diets of alfalfa silage.

If enhanced alfalfa biotypes lead to improvement in cattle growth, it will result from an improvement in NDF digestibility with possible amplification of this benefit via greater DM intake. Presumably, ruminal physical fill limited DMI by steers fed Hi-Gest and HarvXtra, as it did for LegenDairy (Table 4). The compositional benefits in Hi-Gest and HarvXtra were not sufficient to alleviate the physical fill limitation; hence, there was no biotype effect on DMI (Table 4). Without sufficient increases in NDF digestibility and DMI, there was no energetic basis for a steer growth rate benefit. Oba and Allen (1999) recognized the challenging nature of this research topic when they summarized, on the basis of their meta-analysis, that differences among treatments in NDF digestibility were greater when measured in vitro or in situ than when measured in vivo.

There was some evidence that HarvXtra and Hi-Gest 360 biotypes had a more digestible NDF fraction than LegenDairy XHD. The concentration of ADL in HarvXtra was numerically 16.7% less than that of LegenDairy ADL and HarvXtra in vivo alfalfa NDF digestibility was 12.4% greater than for LegenDairy; however, steer DMI and ADG were not affected by biotype. A limitation of these results is that it was based on only one crop year. A second crop year was intended in this project but the yields of HarvXtra and Hi-Gest only allowed a 49-d trial. In addition, metabolizable protein supply from the solely alfalfa diet may have limited steer growth rate. If future research seeks to assess implications for reduced-lignin alfalfa biotypes on cattle performance, differences in cell wall composition and digestibility between the control and modified biotypes should be more disparate than found here for LegenDairy and HarvXtra. In addition, a non-NDF source of RUP should be considered for inclusion in the treatment diets.

## Supplementary Material

txac032_suppl_Supplementary_Appendix

## References

[CIT0001] ADSA-ASAS-PSA. 2020. Guide for care and use of agricultural animals in research and teaching. 4th ed. Champaign, IL.

[CIT0002] Albrecht, K. A., W. F. Wedin, and D. R. Buxton. 1987. Cell-wall composition and digestibility of alfalfa stems and leaves. Crop Sci. 27:735–741. doi:10.2135/cropsci1987.0011183X002700040027x.

[CIT0003] Allen, M. S., and D. R. Mertens. 1988. Evaluating constraints on fiber digestion by rumen microbes. J. Nutr. 118:261–270. doi:10.1093/jn/118.2.261. 2828582

[CIT0004] Ankom Technologies. 2017a. Neutral detergent fiber in feeds – filter bag technique. https://www.ankom.com/sites/default/files/document-files/Method_6_NDF_A200.pdf [accessed October 11, 2020]. Macedon, NY 14502.

[CIT0005] Ankom Technologies. 2017b. Acid detergent fiber in feeds – filter bag technique. https://www.ankom.com/sites/default/files/document-files/Method_5_ADF_A200.pdf [accessed October 11, 2020]. Macedon, NY 14502.

[CIT0006] Ankom Technologies. 2020. Determining acid detergent lignin in Daisy incubator. https://www.ankom.com/sites/default/files/document-files/Method_9_Lignin_in_Daisy_0.pdf (accessed October 11, 2020). Macedon, NY 14502.

[CIT0007] AOAC. 2003. Official methods of analysis of AOAC International. 17th ed. Gaithersburg, MD: Association of Official Analytical Chemists.

[CIT0008] AOCS. 2017. Official methods and recommended practices of the AOCS. 7th ed. Champaign, IL: American Oil Chemists Society.

[CIT0009] Arnold, A. M., K. A. Cassida, K. A. Albrecht, M. H. Hall, D. Min, X. Xu, S. Orloff, D. J. Undersander, E. Santen, and R. M. Sulc. 2019. Multistate evaluation of reduced-lignin alfalfa harvested at different intervals. Crop Sci. 59:1799–1807. doi:10.2135/cropsci2019.01.0023.

[CIT0010] Barnes, R. F. , C. J. Nelson, M. Collins, and K. J. Moore. 2003. Forages: An introduction to grassland agriculture. 6th ed. Vol. I. Ames, IA: Iowa State Press, Blackwell Publishing Company.

[CIT0011] Barros, J., S. Temple, and R. A. Dixon. 2019. Development and commercialization of reduced lignin alfalfa. Curr. Opin Biotechnol. 56:48–54. doi:10.1016/j.copbio.2018.09.003. 30268938

[CIT0012] Broderick, G. A. 1995. Desirable characteristics of forage legumes for improving protein utilization in ruminants. J. Anim. Sci. 73:2760–2773. doi:10.2527/1995.7392760x. 8582869

[CIT0013] Casler, M. D. 1987. In: vitro digestibility of dry matter and cell wall constituents of smooth bromegrass forage. Crop Sci. 27:931–934. doi:10.2135/cropsci1987.0011183X002700050021x.

[CIT0014] Cherney, J. H., S. R. Smith, C. Sheaffer, and D. J. R. Cherney. 2020. Nutritive value and yield of reduced-lignin alfalfa cultivars in monoculture and in binary mixtures with perennial grass. Agron. J. 112:352–367. doi:10.1002/agj2.20045.

[CIT0015] Combs, D. K. 2013. TTNDFD: a new approach to evaluate forages. P. 113–125 In: Proc. 2103 Cornell Nutrition Conf. Dept. Animal Sci., Cornell Univ., Ithaca, NY.

[CIT0016] Dhiman, T. R., C. Cadorniga, and L. D. Satter. 1993. Protein and energy supplementation of high alfalfa silage diets during early lactation. J. Dairy Sci. 76:1945–1959. doi:10.3168/jds.S0022-0302(93)77530-X.

[CIT0017] Donnelly, D. M., J. R. R. Dórea, C. W. Karls, D. M. Schaefer, D. J. Undersander, and D. K. Combs. 2018. Comparing leaf:stem ratio and stem characteristics between reduced lignin and conventional alfalfas over a growth cycle. J. Dairy Sci. 101(E-Suppl. 2):M166.

[CIT0018] Fernandez, A. L. , C. C. Sheaffer, N. E. Tautges, D. H. Putnam, and M. C. Hunter. 2019. Alfalfa, wildlife, and the environment. 2nd ed.. St. Paul, MN: National Alfalfa and Forage Alliance.

[CIT0019] Getachew, G., A. M. Ibanez, W. Pittroff, A. M. Dandekar, M. McCaslin, S. Goyal, P. Reisen, E. J. DePeters, and D. H. Putnam. 2011. A comparative study between lignin down regulated alfalfa lines and their respective unmodified controls on the nutritional characteristics of hay. Anim. Feed Sci. Technol. 170:192–200. doi:10.1016/j.anifeedsci.2011.09.009.

[CIT0020] Getachew, G., E. A. Laca, D. H. Putnam, D. Witte, M. McCaslin, K. P. Ortega, and E. J. DePeters. 2018. The impact of lignin downregulation on alfalfa yield, chemical composition, and in vitro gas production. J. Sci. Food Agric. 98:4205–4215. doi:10.1002/jsfa.8942. 29406620

[CIT0021] Goeser, J. P., and D. K. Combs. 2009. An alternative method to assess 24-h ruminal in vitro neutral detergent fiber digestibility. J. Dairy Sci. 92:3833–3841. doi:10.3168/jds.2008-1136. 19620667

[CIT0022] Goeser, J. P., P. C. Hoffman, and D. K. Combs. 2009. Modification of a rumen fluid priming technique for measuring in vitro neutral detergent fiber digestibility. J. Dairy Sci. 92:3842–3848. doi:10.3168/jds.2008-1745. 19620668

[CIT0023] Grev, A. M., M. S. Wells, D. N. Catalano, K. L. Martinson, J. M. Jungers, and C. C. Sheaffer. 2020. Stem and leaf forage nutritive value and morphology of reduced lignin alfalfa. Agron. J. 112:406–417. doi:10.1002/agj2.20011.

[CIT0024] Grev, A. M., M. S. Wells, D. A. Samac, K. L. Martinson, and C. C. Sheaffer. 2017. Forage accumulation and nutritive value of reduced lignin and reference alfalfa cultivars. Agron. J. 109:2749–2761. doi:10.2134/agronj2017.04.0237.

[CIT0025] Hall, M. B. 2009. Determination of starch, including maltooligosaccharides, in animal feeds: comparison of methods and a method recommended for AOAC collaborative study. J. AOAC Int. 92:42–49. doi:10.1093/jaoac/92.1.42. 19382561

[CIT0026] Huhtanen, P., U. Asikainen, M. Arkkila, and S. Jaakkola. 2007. Cell wall digestion and passage kinetics estimated by marker and in situ methods or by rumen evacuations in cattle fed hay 2 or 18 times daily. Anim. Feed Sci. Technol. 133:206–227. doi:10.1016/j.anifeedsci.2006.05.004.

[CIT0027] Jung, H. G., D. R. Mertens, and A. J. Payne. 1997. Correlation of acid detergent lignin and klason lignin with digestibility of forage dry matter and neutral detergent fiber. J. Dairy Sci. 80:1622–1628. doi:10.3168/jds.S0022-0302(97)76093-4. 9276801

[CIT0028] Karls, C. W. 2020. Reduced-lignin alfalfa digestibility and corn plant botanical part consumption when fed to growing beef steers. M.S. Thesis. Dept of Animal & Dairy Sciences, Univ. Wis.-Madison.

[CIT0029] Kenward, M. G., and J. H. Roger. 2009. An improved approximation to the precision of fixed effects from restricted maximum likelihood. Comput. Stat. Data Anal. 53:2583–2595. doi:10.1016/j.csda.2008.12.013.

[CIT0030] Krizsan, S. J., S. Ahvenjarvi, and P. Huhtanen. 2010. A meta-analysis of passage rate estimated by rumen evacuation with cattle and evaluation of passage rate prediction models. J. Dairy Sci. 93:5890–5901. doi:10.3168/jds.2010-3457. 21094762

[CIT0031] Laboski, C. A. M., and J. B. Peters. 2012. Nutrient application guidelines for field, vegetable, and fruit crops in Wisconsin. Div. Extension, Univ. Wis.-Madison. Madison, WI: Cooperative Extension Publishing.

[CIT0032] Lanzas, C., C. J. Sniffen, S. Seo, L. O. Tedeschi, and D. G. Fox. 2007. A revised CNCPS feed carbohydrate fractionation scheme for formulating rations for ruminants. Anim. Feed Sci. Technol. 136:167–190. doi:10.1016/j.anifeedsci.2006.08.025.

[CIT0033] Lopes, F., K. Ruh, and D. K. Combs. 2015. Validation of an approach to predict total-tract fiber digestibility using a standardized in vitro technique for different diets fed to high-producing dairy cows. J. Dairy Sci. 98:2596–2602. doi:10.3168/jds.2014-8665. 25648802

[CIT0034] Lund, P., M. R. Weisbjerg, and T. Hvelplund. 2007. Digestible NDF is selectively retained in the rumen of dairy cows compared to indigestible NDF. Anim. Feed Sci. Technol. 134:1–17. doi:10.1016/j.anifeedsci.2006.05.016.

[CIT0035] Mertens, D. R., and M. McCaslin. 2008. Evaluation of alfalfa hays with down-regulated lignin biosynthesis. J. Dairy Sci. 91(E-Suppl 1):170.

[CIT0036] National Academies of Sciences, Engineering, and Medicine. 2016. Nutrient requirements of beef cattle. 8th rev. ed. Washington, DC: The National Academies Press.

[CIT0037] NRC. 2001. Nutrient requirements of dairy cattle. 7th rev. ed. Washington, DC: Natl. Acad. Press.

[CIT0038] Oba, M., and M. S. Allen. 1999. Evaluation of the importance of the digestibility of neutral detergent fiber from forage: effects on dry matter intake and milk yield of dairy cows. J. Dairy Sci. 82:589–596. doi:10.3168/jds.S0022-0302(99)75271-9. 10194678

[CIT0039] Peterson, D. M., J. G. P. Bowman, R. L. Endecott, A. L. Mack, and E. C. G. Meccage. 2018. The effects of feeding reduced-lignin alfalfa on growing beef cattle performance: a preliminary study. J. Agric. Stud. 6:87–99. doi:10.5296/jas.v6i2.12872.

[CIT0040] Reddy, M. S. S., F. Chen, G. Shadle, L. Jackson, H. Aljoe, and R. A. Dixon. 2005. Targeted down-regulation of cytochrome P450 enzymes for forage quality improvement in alfalfa (*Medicago sativa* L.). Proc. Natl. Acad. Sci. USA 102:16573–16578. doi:10.1073/pnas.0505749102. 16263933 PMC1283808

[CIT0041] Saxton, A. M. 1998. A macro for converting mean separation output to letter groupings in Proc Mixed. Pages 1243–1246 In Proc. 23rd SAS Users Group Intl., SAS Institute, Cary, NC.

[CIT0042] Schwab, C. G., and G. A. Broderick. 2017. A 100-year review: protein and amino acid nutrition in dairy cows. J. Dairy Sci. 100:10094–10112. doi:10.3168/jds.2017-13320. 29153157

[CIT0043] Sulc, R. M., A. M. Arnold, K. A. Cassida, K. A. Albrecht, M. H. Hall, D. Min, X. Xu, D. J. Undersander, and E. van Santen. 2021. Changes in forage nutritive value of reduced-lignin alfalfa during regrowth. Crop Sci. 61:1478–1487. doi:10.1002/csc2.20366.

[CIT0044] Undersander, D. 2015. Making baleage. Div. Extension, Univ. Wis.-Madison. https://fyi.extension.wisc.edu/forages/files/2015/06/Making-Baleage.pdf [accessed Feb 17, 2022]. Madison, WI: Cooperative Extension Publishing.

[CIT0045] Undersander, D., M. McCaslin, C. Sheaffer, D. Whalen, D. Miller, D. Putnam, and S. Orloff. 2009. Low lignin alfalfa: redefining the yield/quality tradeoff. In: Proceedings, 2009 Western Alfalfa & Forage Conference, December 2-4, 2009, Reno, Nevada. Sponsored by the Cooperative Extension Services of AZ, CA, ID, NV, OR, and WA. Davis: UC Cooperative Extension, Plant Sciences Department, University of California. https://alfalfa.ucdavis.edu/+symposium/2009/files/talks/09WAS23_Undersander_LowLignin.pdf [accessed March 20, 2022].

[CIT0046] Undersander, D., J. E. Moore, and N. Schneider. 2010. Relative forage quality. Focus on forage. 12(6):1–3. https://fyi.extension.wisc.edu/forage/files/2014/01/RFQ-FOF.pdf [accessed. Jan 7, 2021]. Madison, WI: Cooperative Extension Publishing.

[CIT0047] Weakley, D., D. R. Mertens, and M. McCaslin. 2008. Lactating cow responses to alfalfa hays with down-regulated lignin biosynthesis. J. Dairy Sci. 91(E-Suppl 1):170.

